# Association of sleep disorders with subfoveal choroidal thickness in preschool children

**DOI:** 10.1038/s41433-021-01489-y

**Published:** 2021-03-11

**Authors:** Shiya Shen, Xiaoxiao Li, Rui Li, Dan Huang, Xiaoyan Zhao, Xiaohan Zhang, Qingfeng Hao, Qigang Sun, Haohai Tong, Xinyu Zheng, Yelongzi Cao, Shuning Liu, Hui Zhu, Hu Liu

**Affiliations:** 1 Department of Ophthalmology, The First Affiliated Hospital with Nanjing Medical University, Nanjing, China; 2 Department of Child Healthcare, The First Affiliated Hospital with Nanjing Medical University, Nanjing, China; 3 Department of Ophthalmology, Wuxi Children’s Hospital, Wuxi, China; 4 Department of Ophthalmology, Maternal and Child Healthcare Hospital of Yuhuatai District, Nanjing, China; 5 The Fourth School of Clinical Medicine of Nanjing Medical University, Nanjing, China

**Keywords:** Risk factors, Paediatrics, Epidemiology

## Abstract

**Objective:**

To explore the association between sleep disorders and subfoveal choroidal thickness (SFCT) in preschool children.

**Methods:**

In this population-based cross-sectional study, children aged 60–72 months were measured for SFCT using spectral-domain optical coherence tomography (SD-OCT) and for sleep disorders using the Chinese version of Children’s Sleep Habits Questionnaire (CSHQ). Multiple linear regression analyses were performed to assess the association between sleep disorders and SFCT.

**Results:**

A total of 1337 children (mean (SD) age: 66.88 (3.41) months) were included in the analyses. In multivariable linear analysis, a higher total CSHQ score (indicating higher likelihood of sleep disorders) was associated with a thinner subfoveal choroid (beta, −0.070; 95% CI, −0.141 to −0.001; *P* = 0.046). When each of eight CSHQ subscale scores was analysed by the multivariable model, only the Daytime Sleepiness subscale score was negatively associated with the SFCT (beta, −0.115; 95% CI, −0.183 to −0.046; *P* = 0.001). The children with clinically significant daytime sleepiness (*n* = 364, 27.2%) had significantly thinner subfoveal choroid than other children (295.47 vs. 308.52 μm, *P* = 0.007).

**Conclusions:**

Only daytime sleepiness was significantly associated with SFCT in preschool children in this study. The potential relationship between sleep disorders during childhood and children’s ocular development needs further research.

## Introduction

In the past decades, children’s sleep habits have changed substantially, and sleep disorders have become very common [1]. The literature has reported that about 20–60% of preschool children have at least one sleep disorder [1–4]. As an essential physiological phenomenon accompanied by changes in various physiological functions, sleep has significant influence on children’s memory, cognition, behaviour, psychosocial functioning and physical development [5, 6]. Inadequate and disordered sleep has been acknowledged to be associated with numerous impairments and diseases, such as injury [7], depression, anxiety [8], autism, attention-deficit/hyperactivity disorder [9], weight gain and obesity [10], diabetes, heart disease and dementia [11, 12].

The choroid is a heterogeneous tissue consisting of blood vessels and stroma. As the main source of ocular blood flow, its structural and functional integrity is important for ocular development and health. Changed choroidal thickness has been found in various diseases [13, 14]. In addition, choroidal thickness has been shown to be associated with sleep disorders in adult. A meta-analysis suggested that subfoveal choroidal thickness (SFCT) was significantly reduced in adult patients with different severities of obstructive sleep apnea syndrome (OSAS) [15]. The mechanism of reduced choroidal thickness in OSAS might be related to hypoxia induced by OSAS, but has not been verified yet.

The effect of sleep disorders on children’s choroidal thickness remains unknown. Previous studies showed that changes in choroidal thickness is closely associated with the development of myopia and myopic maculopathy [16–20]. In addition, sleep disorders were also found to be associated with myopia in children [21]. Therefore, we speculate that there might be a relationship between sleep disorders and choroidal thickness in children. To test this hypothesis, we analysed the data from the population-based Nanjing Eye Study (NES) to assess the association of sleep disorders with SFCT in preschool children aged 60–72 months.

## Materials and methods

### Study population

The study participants are from the population-based Nanjing Eye Study (NES). The details of NES have been reported before [22–24]. In brief, all children born in Yuhuatai District, Nanjing, China between September 2011 and August 2012, and entering kindergartens in Yuhuatai District, were invited to participate in the NES. All participants underwent a comprehensive eye examination annually since 2015. The data presented in this paper were obtained in 2017, when these children were 60–72 months old. Children with history of or ongoing ocular diseases, history of ocular surgery, or congenital malformations were excluded from this study. The study was approved by the Ethics Committee of The First Affiliated Hospital with Nanjing Medical University and followed the tenets of the Declaration of Helsinki. Written informed consent was obtained from the parents or legal guardians of all participants. Oral assent was obtained from all children right before the examination.

### Ocular and anthropometric examinations

A comprehensive eye examination for all participants was performed by a team composed of six trained ophthalmologists and four optometrists, including visual acuity, anterior segment, posterior segment, refraction, stereoacuity test, ocular alignment and motility, ocular biometric parameters, intraocular pressure, accommodative response and optical coherence tomography. Lenstar LS900 (Haag-Streit Koeniz, Switzerland) was used to measure ocular biometric parameters under non-cycloplegic conditions, including axial length (AL), corneal radius of curvature (CR), central corneal thickness, anterior chamber depth, lens thickness. All these measurements were taken five times and then averaged. Retinoscopy was performed after cycloplegia to measure refraction. One drop of topical 1.0% cyclopentolate (Cyclogyl, Alcon Pharmaceuticals) was administered to each eye twice at a 5 min interval. After 15 min, a third drop of cyclopentolate was administered if the pupil size was <6 mm or the pupillary light reflex was still present. The spherical equivalent (SE) was calculated as the sphere power plus half of the cylinder power. Anthropometric parameters included height and weight, which were measured without shoes and heavy clothing. Body mass index was defined as body weight divided by the square of body height and was expressed in units of kg/m^2^.

**Fig. 1:**
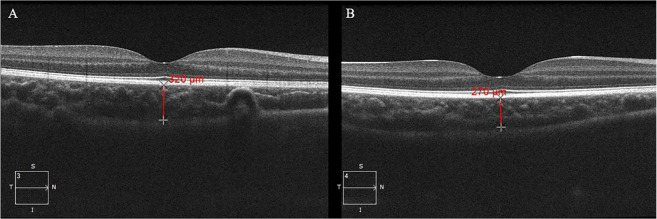
Choroidal thickness measurement. Subfoveal choroidal thickness (red line) was defined as the distance from the outer border of the retinal pigment epithelium to the inner border of the sclera running through the centre of the fovea. **A** Subfoveal choroidal thickness in a normal child. **B** Subfoveal choroidal thickness in a child with daytime sleepiness.

The choroid was imaged using spectral-domain optical coherence tomography (SD-OCT) (Cirrus HD-OCT 5000; Carl Zeiss Meditec, Inc., Dublin, CA, USA) with the enhanced depth imaging system under a cross line scan pattern focused on the fovea. Each line was composed of 8 B-scans consisting of 1024 A-scans. Each scan was required to have a scan signal intensity ≥6 and captured in a similar time period within a day without pupil dilation. SFCT was defined as the distance from the outer border of the retinal pigment epithelium to the inner border of the sclera running through the centre of the fovea, and was measured by a trained examiner using a manual calliper of the device software (Fig. 1). The centre of the fovea was identified on the scan line with the deepest foveal depression and a pronounced specular bottom reflex. Choroidal images and measurements were obtained from both eyes. Intra-grader reliability for the SFCT was high with intraclass correlation of 0.963 based on 300 eyes with SFCT measured twice.

### Questionnaires

A comprehensive questionnaire was distributed to a parent of each participant to collect information, mainly including history of pregnancy and birth, home environment, daily activities, and sleep disorders. Children’s daily activities per week were divided into five activities: (1) outdoor activities; (2) studying and reading for school assignments; (3) reading for pleasure; (4) using a computer or electronic products/playing video games; (5) watching television. The dioptre-hour (Dh) was defined as: Dh = 3 × (hours of studying and reading for school + hours of reading for pleasure) + 2 × (video game/computer/electronic product hours) + 1 × (hours of watching television) [25, 26]. The well-validated Chinese version of Children’s Sleep Habits Questionnaire (CSHQ) was used to assess sleep disorders [27–29]. This questionnaire includes total of 33 items, covering 8 subscales: Bedtime Resistance, Sleep Onset Delay, Sleep Duration, Sleep Anxiety, Night Wakings, Parasomnias, Sleep Disordered Breathing and Daytime Sleepiness. Parents were asked to rate the frequency for each item based on the observation of the children in the past recent week or a typical week on a 3-point Likert Scale: “usually” (5–7 times per week), “sometimes” (2–4 times per week) and “rarely” (0–1 time per week). The subscale scores and total CSHQ score were calculated following standard scoring algorithm. The total CSHQ score ranges from 33 to 99, with higher total score indicating greater sleep problems [30]. A total score > 41 has been suggested to identify children with global sleep disorders [31, 32]. For each of 8 subscale scores, a subscale score > 1 SD above the mean subscale score of the published control sample was considered to be clinically significant [30, 33].

In addition, evening bedtime and wake-up time for weekdays and weekends were also collected. The calculation formula for average sleep duration was calculated as follows: [(weekday sleep duration × 5) + (weekend sleep duration × 2)]/7 [10].

### Statistical analysis

Data analyses were performed using the IBM Statistical Package for the Social Sciences statistics for Windows (V.13.0, Armonk, NY, USA). For eye-specific measurement, only data from the right eye of each participant were included for analysis. Continuous data were presented as mean and standard deviation (SD). Independent two sample T-test was used to compare means and chi-squared test was used to compare proportions. Univariable linear regression model was used to evaluate the association between each candidate factors and SFCT. Factors with *P* value < 0.20 in univariable analysis were included in the multivariable linear regression for backward variable selection, and the final multivariable regression model evaluating the association of overall total CSHQ score and SFCT was adjusted by risk factors with *p* < 0.05 along with age and gender. Similar analyses were performed for evaluating association of each subscale of CSHQ score with SFCT by including the same risk factors as model for evaluating total CSHQ score. Standardised regression coefficients from the regression models are presented with 95% confidence intervals (95% CI). Two-sided *P* values < 0.05 were considered statistically significant.

## Results

Among 2300 eligible children, 1920 (response rate 83.48%) participated in the study and 583 were excluded because of incomplete ocular examinations or poor quality of OCT images. The children excluded were not different from the included children in age, gender or other characteristics (see Supplementary sTable 1). Among 1337 children included in the analysis, the mean (SD) age was 66.88 (3.41) months and 45.5% (*n* = 609) were girls. The mean (SD) SFCT was 307.41 (63.42) μm. The mean (SD) sleep duration was 9.99 (0.64) h per day. Among all participants, the CSHQ total score ranged 33 to 70, with mean (SD) of CSHQ total score 47.33 (5.13). 88.8% (1187/1337) children had total CSHQ score above 41 (the cut-off for global sleep disorder). The characteristics of the participants were shown in Table 1.

**Table 1:** Characteristics of the 1337 participants.

Parameters	Mean (SD)/number (%)
Age, *m*	66.88 (3.41)
Gender (girls), %	609 (45.5)
SFCT, μm	307.41 (63.42)
Height, cm	111.27 (7.57)
Weight, kg	19.91 (4.08)
Body mass index, kg/m^2^	15.94 (1.80)
Birthweight, kg	3.32 (0.54)
Spherical equivalent, D	1.18 (0.93)
Axial length, mm	22.49 (0.71)
Central corneal thickness, μm	541.44 (31.25)
Anterior chamber depth, mm	2.82 (0.25)
Lens thickness, mm	3.69 (0.22)
Corneal radius of curvature, mm	7.83 (0.25)
Outdoor activities, h/w	15.55 (7.85)
Dioptre-hour, h/w	75.94 (68.00)
Sleep duration, h/d	9.99 (0.64)
CSHQ score	47.33 (5.13)
Father with myopia, %	472 (35.3)
Mother with myopia, %	515 (38.5)
Father with high school or below, %	682 (51.0)
Mother with high school or below, %	710 (53.1)
Pre-term history, %	64 (4.8)
Maternal second-hand smoking exposure when pregnancy, %	189 (14.1)

*SFCT* subfoveal choroidal thickness, *CHSQ* score the score of Children’s Sleep Habits Questionnaire.

Associations of SFCT and its candidate risk factors were shown in Table 2. In univariable linear regression, SFCT was significantly associated with gender, SE, AL and CR (*P* < 0.05), and marginally associated with total CSHQ scores (*P* = 0.05). In multivariable linear regression, SFCT was significantly associated with total CSHQ score (*P* = 0.046) after adjusting for age, gender, height, birthweight and AL. The standardised regression coefficient from the multivariable regression model was −0.070 (95% CI: −0.141 to −0.001), suggesting more sleep disorder was associated with thinner SFCT.

**Table 2:** Univariable and multivariable analyses for associations between all potential factors and SFCT.

Parameters	Univariable analysis		Multivariable analysis^a^	
	B^b^ (95% CI)	*P*	B (95% CI)	*P*
Age, *m*	0.039 (−0.019, 0.096)	0.19	0.042 (−0.030, 0.111)	0.26
Gender				
Boys	Ref		Ref	
Girls	0.091 (0.034, 0.149)	**0.002**	0.056 (−0.019, 0.129)	0.14
Height, cm	−0.059 (−0.118, 0.001)	0.06	−0.094 (−0.165, −0.020)	**0.01**
Weight, kg	−0.047 (−0.105, −0.012)	0.12		
Body mass index, kg/m^2^	−0.025 (−0.084, 0.034)	0.41		
Birthweight, kg	0.055 (−0.012, 0.120)	0.11	0.101 (0.028, 0.165)	**0.006**
Spherical equivalent, D	0.077 (0.020, 0.139)	**0.009**		
Axial length, mm	−0.171 (−0.232, −0.116)	**<0.001**	−0.155 (−0.236, −0.078)	**<0.001**
Central corneal thickness, μm	−0.044 (−0.099, 0.013)	0.14		
Anterior chamber depth, mm	−0.006 (−0.064, 0.052)	0.84		
Lens thickness, mm	0.035 (−0.024, 0.095)	0.24		
Corneal radius of curvature, mm	−0.136 (−0.195, −0.080)	**<0.001**		
Outdoor activities, h/w	0.029 (−0.039, 0.096)	0.40		
Dioptre-hour, h/w	−0.017 (−0.080, 0.047)	0.62		
Sleep duration, h/d	−0.052 (−0.120, 0.015)	0.13		
CSHQ score	−0.066 (−0.136, 0.001)	**0.05**	−0.070 (−0.141, −0.001)	**0.046**
Father with myopia				
Yes	0.002 (−0.064, 0.069)	0.95		
No	Ref			
Mother with myopia				
Yes	0.006 (−0.061, 0.073)	0.86		
No	Ref			
Father education level				
High school or below	−0.044 (−0.111, 0.023)	0.20		
University degree or above	Ref			
Mother education level				
High school or below	−0.035 (−0.101, 0.032)	0.31		
University degree or above	Ref			
Pre-term history				
Yes	−0.064 (−0.130, 0.003)	0.06		
No	Ref			
Maternal second-hand smoking exposure when pregnancy				
Yes	−0.010 (−0.078, 0.057)	0.77		
No	Ref			

Bold values indicate statistical significance *p* ≤ 0.05.

*SFCT* subfoveal choroidal thickness, *CHSQ* score the score of Children’s Sleep Habits Questionnaire, *Ref* reference group.

^a^The multivariable model was adjusted for age, gender, AL, height and birthweight.

^b^The regression coefficient B is standardised regression coefficient.

When each of eight CSHQ subscale scores was analysed using the multivariable model separately instead of total CSHQ score, only the Daytime Sleepiness subscale score was found to be negatively associated with choroidal thickness (beta, −0.115, *P* = 0.001, Table 3) after adjusting for age, gender, height, AL and birthweight. Approximately 27.2% (364/1337) children scored more than 1 SD above the published normative mean in the Daytime Sleepiness subscale (i.e. above 12.44) and were considered to have clinically significant daytime sleepiness. In children with clinically significant daytime sleepiness, SFCT was 13.05 μm thinner compared to children without this problem (295.47 μm vs. 308.52 μm, *P* = 0.007, Table 4), after adjusting for age, gender, height, AL and birthweight.

**Table 3:** The associations between CSHQ subscale scores and SFCT.

Subscale scores	Mean (SD)	Univariable analysis		Multivariable analysis^a^	
		B^b^ (95% CI)	*P*	B (95% CI)	*P*
Bedtime resistance	10.91 (1.97)	−0.038 (−0.104, 0.029)	0.27		
Sleep onset delay	2.21 (0.75)	0.017 (−0.049, 0.084)	0.61		
Sleep duration	5.85 (1.20)	−0.007 (−0.074, 0.060)	0.85		
Sleep anxiety	6.42 (1.98)	−0.064 (−0.130, 0.003)	0.06		
Night wakings	3.32 (0.69)	−0.044 (−0.116, 0.024)	0.20		
Parasomnias	7.83 (1.17)	−0.009 (−0.080, 0.061)	0.80		
Sleep disordered breathing	3.35 (0.73)	0.028 (−0.039, 0.096)	0.41		
Daytime sleepiness	11.30 (2.10)	−0.098 (−0.166, −0.031)	**0.004**	−0.115 (−0.183, −0.046)	**0.001**

Bold values indicate statistical significance *p* ≤ 0.05.

*SFCT* subfoveal choroidal thickness, *CHSQ* score the score of Children’s Sleep Habits Questionnaire.

^a^The multivariable model was adjusted for age, gender, AL, height and birthweight.

^b^The regression coefficient B is standardised regression coefficient.

**Table 4:** The associations between clinically significant subscales and SFCT.

Subscales	Cut-off values^a^	Clinically significant	Number	Univariable analysis		Multivariable analysis^b^		
				B^c^ (95% CI)	*P*	Adjusted mean (95% CI)	B (95% CI)	*P*
Bedtime resistance	8.95	Yes	1197	−0.042 (−0.107, 0.025)	0.23			
		No	140	Ref				
Sleep onset delay	1.78	Yes	1072	0.038 (−0.029, 0.105)	0.27			
		No	265	Ref				
Sleep duration	4.34	Yes	1182	−0.017 (−0.083, 0.050)	0.62			
		No	155	Ref				
Sleep anxiety	6.34	Yes	578	−0.059 (−0.126, 0.008)	0.09			
		No	759	Ref				
Night wakings	4.40	Yes	118	−0.055 (−0.130, 0.013)	0.11			
		No	1219	Ref				
Parasomnias	9.36	Yes	116	0.015 (−0.054, 0.083)	0.67			
		No	1221	Ref				
Sleep disordered breathing	3.87	Yes	325	0.035 (−0.032, 0.102)	0.31			
		No	1012	Ref				
Daytime sleepiness	12.44	Yes	364	−0.078 (−0.144, −0.011)	**0.02**	295.47 (287.42, 303.51)	−0.094 (−0.159, −0.025)	**0.007**
		No	973	Ref		308.52 (303.52, 313.51)		

Bold values indicate statistical significance *p* ≤ 0.05.

*SFCT* subfoveal choroidal thickness, *Ref* reference group.

^a^Cut-off value is 1 SD above the mean score of the published control sample.

^b^The multivariable model was adjusted for age, gender, AL, height and birthweight.

^c^The regression coefficient B is standardised regression coefficient.

## Discussion

Our study found that higher total CSHQ score (indicating higher likelihood of more sleep problems) was associated with thinner subfoveal choroid in children aged 60–72 months after adjusting for other confounding factors. More specifically, among the eight CSHQ subscale scores, only the Daytime Sleepiness subscale score was negatively associated with SFCT. Children with clinically significant daytime sleepiness had 13.05 μm thinner subfoveal choroid than these without this sleep disorder.

Our study used the CSHQ to evaluate the sleep habits of participants. The CSHQ is one of the most common used instruments to screen sleep disorders among children aged 4–10 years [30]. It is designed on the basis of the International Classification of Sleep Disorders: Diagnostic and Coding Manual and can target common sleep disorder symptoms in children [34]. The validity of this questionnaire has been evaluated in more than 200 studies all over the world, and it has been cited in over 600 articles and translated into many languages [28, 35]. The reliability and validity of the Chinese version of the CSHQ we used have been verified in several studies [27–29]. Thus, we believe that CSHQ can provide valid data of sleep habits of our study participants for evaluating its association with SFCT. In this study, 88.8% participants had sleep disorder (CSHQ score > 41), which is consistent with a previous study conducted in Chinese preschool children using the CSHQ questionnaire [31].

Of 8 subscales of the CSHQ, we only found daytime sleepiness was associated with SFCT. Daytime sleepiness is an easily ignored sleep disorder in children. According to a community-based research in children aged 5–12 years, the prevalence of parent or teacher-reported daytime sleepiness was nearly 15% [36]. In our study, about 27% children had clinically significant daytime sleepiness measured by CSHQ. The primary cause of daytime sleepiness among children is insufficient sleep, which is often related with poor sleep hygiene [37]. Besides, some diseases may result in daytime sleepiness, such as OSAS, periodic limb movement, restless leg syndrome, narcolepsy, Kleine–Levin syndrome and so on [5]. Previous studies suggested that children with daytime sleepiness were more likely to have impairments in functional areas, including behaviour, mood, and performance [38]. Our study provides evidence that daytime sleepiness may be related with changes in ocular choroidal structure in children. Although insufficient sleep is the main cause of daytime sleepiness, we failed to find associations between sleep duration and SFCT. This may be due to the fact that sleep duration reported by parents is just the period from bedtime to time to get up and does not necessarily represent the actual amounts of sleep in children. Delayed sleep onset latency, frequent night awakenings and other factors that may impair sleep quality can also cause insufficient sleep.

Our study found that in children with clinically significant daytime sleepiness, SFCT was 13.05 μm thinner compared to children without this problem. This magnitude of difference in SFCT is clinically significant compared with previous studies, which found that one dioptre toward myopia was associated with a decrease of 10 μm in central foveal choroidal thickness and child exposure to second-hand smoking was associated with a decrease of 8.3 μm in central foveal choroidal thickness [39, 40]. Therefore, more attention should be paid to the sleep hygiene in children.

In adults, the change of choroidal thickness was only explored in one kind of sleep disorders, that is, OSAS. In a meta-analysis performed to evaluate the choroidal thickness changes in OSAS, SFCT was significantly reduced compared with normal controls, with mean difference of −8.06 to −53.72 μm for mild to severe OSAS [15]. Although the exact mechanism for this association remains unclear, it is speculated that intermittent airway obstruction in OSAS causes recurrent hypoxia and reperfusion, which leads to oxidative stress and inflammation, vascular endothelium damage, impaired responsiveness to vasodilator agents, and activated sympathetic system [41]. The series of changes result in decreased choroidal blood flow, and therefore reduced choroidal thickness. As mentioned above, OSAS can cause daytime sleepiness in children, so it can be one of the underlying mechanisms for the thinner SFCT in children with daytime sleepiness.

As the prevalence of OSAS is only from 1.2 up to 5% in children [42, 43], there must be other reasons for explaining our significant association between sleep disorders and SFCT. Most daytime sleepiness is caused by insufficient sleep, associated with poor sleep hygiene such as irregular sleep schedules, electronics use before bed, and vigorous play before bed [44]. These behaviours can interfere with circadian sleep-wake rhythm among children and contribute to irregular secretion of melatonin [12]. As a neurohormone, melatonin can act directly on many ocular structures to mediate various circadian rhythms and physiological processes in the eye [45]. Melatonin receptors are also present in the choroid [46]. It has been found in animal experiments that systemic administration of melatonin resulted in significant changes in choroidal thickness [47]. Therefore, the association between choroidal thickness and daytime sleepiness may be due to changes in melatonin secretion, which affects the normal growth of the choroid.

We did not find the association between the sleep disordered breathing subscale and SFCT. This might be due to, among children who scored high in the sleep disordered breathing subscale, only a small number (1 ‘usually’, 2 ‘sometimes’) had the problem of stopping breathing in sleep and were likely to have OSAS. Besides, the sleep disordered breathing might interfere less with circadian rhythm than the daytime sleepiness. In addition, other subscales of CSHQ were not associated with SFCT in our study, which might be because of their not close relationship with OSAS and circadian rhythm. The borderline association between the overall sleep questionnaire and SFCT might be due to that no relationship between the seven subscales and SFCT was found. However, further research is needed to clarify why only daytime sleepiness subscale was related with SFCT.

There are few studies for choroidal thickness in preschool children younger than 6 years old, partially due to the difficulty in obtaining good OCT images from these young children. To obtain reliable measurement of choroidal thickness, this study only included participants with choroidal OCT images of high quality in a large population-based sample. In addition, all choroidal images in this study were obtained before pupil dilation and in a similar time period within a day, which further eliminated possible factors affecting choroidal thickness [48, 49]. Besides, we included more than 20 possible confounders in the linear regression analyses, which raises the accuracy of our results.

Our study still has some limitations. First, sleep disorders were assessed by parents through questionnaires, which may have recall bias. Objective measures of children’s sleep behaviour, like polysomnography and actigraphy, may produce highly reliable and valid data. However, the cost, time, and effort associated with these measures make them difficult to implement in epidemiological investigations like our study. Second, about 16.5% children refused to participate in our study and we excluded some participants from analysis because of incomplete ocular examinations or poor quality of OCT images, which may lead to the biased estimate of association. Third, we only measured SFCT in this study, and the associations between sleep disorders and choroidal thickness in other sectors are unknown. Nevertheless, complex scan pattern needed to collect the choroidal images in different sectors is time-consuming, thus difficult to carry out in preschool children because they cannot keep fixation for a long time. Fourth, like other cross-sectional studies, we could not make any causal inference for the association between daytime sleepiness and SFCT. In addition, only daytime sleepiness subscale was found to be related with SFCT among the 8 subscales and the association between the overall sleep questionnaire score and SFCT was marginal. The results of this exploratory study need more replications and verifications. Fifth, 4.8% of our participants had a pre-term history, which might have an influence on the results. However, the association between CSHQ score and SFCT still existed after including pre-term history in the multivariable model (see sTable 2). Finally, the association between daytime sleepiness and SFCT found in our study might be due to the possible relationship between sleep disorders and myopia (characterised by decreased choroidal thickness), which was reported in several previous studies [21, 50]. However, AL and SE were adjusted for in the multivariable analysis (see Table 2). Besides, we failed to find an association between sleep disorders and SE or AL (see sTables 3,  4), which was consistent to the findings in recent studies [51, 52]. More research may be warranted to clarify the potentially triangular relationship between sleep disorders, myopia, and choroidal thickness.

## Conclusions

In this cross-sectional population-based study, we found that daytime sleepiness was significantly associated with SFCT in children aged 60–72 months after adjusting for confounding factors. Children with clinically significant daytime sleepiness had 13.05 μm thinner SFCT compared to children without this sleep disorder. Although this finding cannot prove a causative relationship and the exact mechanism remains unknown, this study provides important information that, daytime sleepiness, a common sleep disorder in modern society, is associated with altered ocular health in early childhood.

## Summary

### What was known before

- Sleep disorders were very common in children, and have been acknowledged to be associated with numerous impairments and diseases.
- Choroid is the main source of ocular blood flow and is important for ocular development and health. Adults with obstructive sleep apnoea syndrome (OSAS) have been reported to have reduced SFCT.
- The effect of sleep disorders on children’s choroidal thickness remains unknown.

### What this study adds

- We found that more daytime sleepiness in preschool children was associated with subfoveal choroidal thinning, which is independent of the known factors that influence choroidal thickness.
- Sleep disorder is associated with altered ocular health in early childhood.
- Daytime sleepiness warrants early recognition and management.

## Supplementary information

sTable 1. Comparison between included and excluded children

sTable 2. The association between CSHQ score and SFCT after including pre-term history in the multivariable model

sTable 3. Univariable and multivariable analysis for associations between sleep disorders and SE

sTable 4. Univariable and multivariable analysis for associations between sleep disorders and AL
