# Supplementary material for: Association of sleep disorders with subfoveal choroidal thickness in preschool children
Source: Eye (Lond). 2021 Mar 11;36(2):448–56. doi: 10.1038/s41433-021-01489-y (PMC8807704; doi:10.1038/s41433-021-01489-y)
Supplement: Supplementary file 1 — sTable 1. Comparison between included and excluded children [file 41433_2021_1489_MOESM1_ESM.docx]

| **sTable 1**. Comparison between included and excluded children | | | |
| --- | --- | --- | --- |
|  | **Included, n=1337** | **Excluded, n=583** |  |
| **Parameters** | **Mean (SD) / Number (%)** | **Mean (SD) / Number (%)** | **P^*^** |
| **Age, m** | 66.88 (3.41) | 66.73 (3.32) | 0.36 |
| **Gender (Girls), %** | 609 (45.5) | 260 (44.6) | 0.70 |
| **Height, cm** | 111.27 (7.57) | 110.63 (7.70) | 0.10 |
| **Weight, kg** | 19.91 (4.08) | 19.62 (3.82) | 0.15 |
| **Body mass index, kg/m2** | 15.94 (1.80) | 15.91 (1.67) | 0.79 |
| **Birthweight, kg** | 3.33 (0.54) | 3.36 (0.48) | 0.30 |
| **SFCT, μm** | 307.41 (63.42) | 312.91 (66.20) | 0.12 |
| **CSHQ score** | 47.33 (5.13) | 47.38 (5.20) | 0.86 |
| SFCT, subfoveal choroidal thickness; CHSQ score, the score of Children's Sleep Habits Questionnaire | | | |
| ^*^P-value was calculated using independent-sample t-test. | | | |
