# Supplementary material for: Association of sleep disorders with subfoveal choroidal thickness in preschool children
Source: Eye (Lond). 2021 Mar 11;36(2):448–56. doi: 10.1038/s41433-021-01489-y (PMC8807704; doi:10.1038/s41433-021-01489-y)
Supplement: Supplementary file 2 — sTable 2. The association between CSHQ score and SFCT after including pre-term history in the multivariable model [file 41433_2021_1489_MOESM2_ESM.docx]

| **sTable 2.** The association between CSHQ score and SFCT after including pre-term history in the multivariable model | | | | |
| --- | --- | --- | --- | --- |
| **Parameters** | **Univariable Analysis** | | **Multivariable Analysis** | |
|  | **B^†^ (95% CI)** | **P** | **B^†^ (95% CI)** | **P** |
| **Age, m** | 0.039 (-0.019. 0.096) | 0.19 | 0.041 (-0.031, 0.113) | 0.26 |
| **Gender** |  |  |  |  |
| Boys | Ref |  | Ref |  |
| Girls | 0.091 (0.034, 0.149) | **0.002** | 0.052 (-0.023, 0.127) | 0.17 |
| **Height, cm** | -0.059 (-0.118, 0.001) | 0.06 | -0.097 (-0.171, -0.024) | 0.09 |
| **Birthweight, kg** | 0.055 (-0.012, 0.120) | 0.11 | 0.090 (0.018, 0.163) | **0.015** |
| **Axial length, mm** | -0.171 (-0.232, -0.116) | **<0.001** | -0.157 (-0.238, -0.077) | **<0.001** |
| **Preterm history** |  |  |  |  |
| Yes | -0.064 (-0.130, 0.003) | 0.06 | -0.032 (-0.102, 0.039) | 0.375 |
| No | Ref |  | Ref |  |
| **CSHQ score** | -0.066 (-0.136, 0.001) | **0.05** | -0.072 (-0.143, -0.001) | **0.046** |
| SFCT, subfoveal choroidal thickness; CHSQ score, the score of Children's Sleep Habits Questionnaire; Ref, reference group. | | | | |
| ^†^The regression coefficient B is standardized regression coefficient. | | | | |
