# Supplementary material for: Association of sleep disorders with subfoveal choroidal thickness in preschool children
Source: Eye (Lond). 2021 Mar 11;36(2):448–56. doi: 10.1038/s41433-021-01489-y (PMC8807704; doi:10.1038/s41433-021-01489-y)
Supplement: Supplementary file 3 — sTable 3. Univariable and multivariable analysis for associations between sleep disorders and SE [file 41433_2021_1489_MOESM3_ESM.docx]

| **sTable 3**. Univariable and multivariable analysis for associations between sleep disorders and SE | | | | |
| --- | --- | --- | --- | --- |
| **Sleep disorders** | **Univariable Analysis** | | **Multivariable Analysis^*^** | |
|  | **B^†^ (95% CI)** | **P** | **B (95% CI)** | **P** |
| **Bedtime Resistance** | -0.026 (-0.088, 0.035) | 0.402 | -0.006 (-0.057, 0.045) | 0.824 |
| **Sleep Onset Delay** | -0.020 (-0.082, 0.042) | 0.532 | 0.013 (-0.037, 0.063) | 0.613 |
| **Sleep Duration** | -0.025 (-0.087, 0.037) | 0.425 | -0.007 (-0.059, 0.044) | 0.786 |
| **Sleep Anxiety** | -0.046 (-0.108, 0.016) | 0.142 | -0.033 (-0.083, 0.016) | 0.188 |
| **Night Wakings** | 0.001 (-0.061, 0.063) | 0.982 | -0.007 (-0.059, 0.045) | 0.787 |
| **Parasomnias** | -0.037 (-0.099, 0.025) | 0.238 | -0.001 (-0.053, 0.052) | 0.981 |
| **Sleep Disordered Breathing** | 0.076 (0.014, 0.138) | **0.016** | 0.026 (-0.025, 0.077) | 0.314 |
| **Daytime Sleepiness** | 0.014 (-0.048, 0.076) | 0.659 | 0.002 (-0.049, 0.053) | 0.943 |
| ^*^The multivariable model was adjusted for age, gender, height, weight, parental myopia, time for outdoor activities and dioptre-hour. | | | | |
| ^†^The regression coefficient B is standardized regression coefficient. | | | |  |
