# Supplementary material for: Association of sleep disorders with subfoveal choroidal thickness in preschool children
Source: Eye (Lond). 2021 Mar 11;36(2):448–56. doi: 10.1038/s41433-021-01489-y (PMC8807704; doi:10.1038/s41433-021-01489-y)
Supplement: Supplementary file 4 — sTable 4. Univariable and multivariable analysis for associations between sleep disorders and AL [file 41433_2021_1489_MOESM4_ESM.docx]

| **sTable 4**. Univariable and multivariable analysis for associations between sleep disorders and AL | | | | |
| --- | --- | --- | --- | --- |
| **Sleep disorders** | **Univariable Analysis** | | **Multivariable Analysis^*^** | |
|  | **B^†^ (95% CI)** | **P** | **B (95% CI)** | **P** |
| **Bedtime Resistance** | -0.013 (-0.075, 0.049) | 0.405 | -0.012 (-0.043, 0.018) | 0.43 |
| **Sleep Onset Delay** | -0.007 (-0.069, 0.055) | 0.833 | 0.020 (-0.010, 0.049) | 0.198 |
| **Sleep Duration** | -0.036 (-0.089, 0.026) | 0.258 | 0.002 (-0.029, 0.033) | 0.898 |
| **Sleep Anxiety** | -0.006 (-0.068, 0.057) | 0.86 | -0.026 (-0.056, 0.004) | 0.087 |
| **Night Wakings** | 0.039 (-0.023, 0.101) | 0.222 | -0.015 (-0.046, 0.016) | 0.343 |
| **Parasomnias** | 0.026 (-0.036, 0.088) | 0.413 | -0.001 (-0.033, 0.030) | 0.941 |
| **Sleep Disordered Breathing** | -0.016 (-0.078, 0.046) | 0.616 | -0.022 (-0.052, 0.008) | 0.157 |
| **Daytime Sleepiness** | 0.008 (-0.054, 0.070) | 0.805 | -0.007 (-0.037, 0.023) | 0.655 |
| ^*^The multivariable model was adjusted for age, gender, height, weight, parental myopia, time for outdoor activities and dioptre-hour. | | | | |
| ^†^The regression coefficient B is standardized regression coefficient. | | | | |
